# Characterization of clinical *Vibrio parahaemolyticus* strains in Zhoushan, China, from 2013 to 2014

**DOI:** 10.1371/journal.pone.0180335

**Published:** 2017-07-05

**Authors:** Hongling Wang, Xiaoyang Tang, Yi-Cheng Su, Jiabei Chen, Jianbo Yan

**Affiliations:** 1 Key Laboratory of Health Risk Factors for Seafood of Zhejiang Province, Zhoushan, Zhejiang, China; 2 Zhoushan Municipal Center for Disease Control and Prevention, Zhoushan, Zhejiang, China; 3 Food Safety Research Center, Central Research Institute, MasterKong Holding, Shanghai, China; 4 Seafood Research and Education Center, Oregon State University, Astoria, Oregon, United States of America; Ross University School of Veterinary Medicine, SAINT KITTS AND NEVIS

## Abstract

*Vibrio parahaemolyticus* is recognized as major cause of foodborne illness of global public health concern. This study collected 107 strains of *V*. *parahaemolyticus* during active surveillance of diarrheal diseases in hospitals in Zhoushan during 2013 to 2014 and investigated their serotypes, virulence genes (*tdh*, *trh*, and *orf8*), antimicrobial resistance, and genotypes. The dominant serotypes of the 107 clinical strains were O3:K6, O4:K8, and O4:KUT with 87.9% and 3.7% of the strains carrying the virulence genes *tdh* and *trh*, respectively. Molecular typing by pulsed-field gel electrophoresis indicated divergence among the clinical strains. Most isolates were sensitive to the common antimicrobial agents used against the *Vibrio* species except ampicillin. We conclude that continuous surveillance of *V*. *parahaemolyticus* in diarrhea patients is a public health priority and is useful for conducting risk assessment of foodborne illnesses caused by *V*. *parahaemolyticus*.

## Introduction

*Vibrio parahaemolyticus* is a gram-negative halophilic human pathogen that naturally occurs in marine or estuarine environments, which is frequently isolated from a variety of seafood, such as shrimp, oyster, and fish [1–3]. Infections caused by *V*. *parahaemolyticus* can cause acute human gastroenteritis with major symptoms of headache, abdominal pain, and diarrhea, and in some cases, wound infection and septicemia [4–6]. This foodborne pathogen is considered the leading cause of seafood-derived illness in many countries around the world, including the United States, Thailand, Malaysia, Japan, Korea, and China [6–9]. In China, a total of 322 gastroenteritis outbreaks involving 9,041 illnesses and 3,948 hospitalizations due to *V*. *parahaemolyticus* infection were reported from 2003 to 2008 [10].

Zhoushan Fishery is the largest fishery in China. The output of aquatic products in Zhoushan is around 1.3 million tons annually, meanwhile, the offshore ocean fishing output account formore than one tenth of the total output in China [11]. Many citizens of Zhoushan in China regularly consume raw or undercooked seafood. Therefore, *V*. *parahaemolyticus* is a common regional pathogen, which causes more than 70% of the food-poisoning cases in Zhoushan [12]. Despite the high risk of *V*. *parahaemolyticus*, information regarding the prevalence or molecular epidemiology of this pathogen is not readily available.

The main objective of the present study was to investigate *V*. *parahaemolyticus* strains collected from stool specimens of outpatients with diarrheal disease in Zhoushan hospitals and to analyze their serotypes, virulence genes, genotypic traits, and antimicrobial resistance. The investigation will provide vital information to government agencies for performing risk assessments of *V*. *parahaemolyticus* infections.

## Materials and methods

### Bacterial isolates

A total of 107 clinical *V*. *parahaemolyticus* isolates were used in the present study. They were collected from the stool specimens of 107 sporadic diarrhea outpatients from the active surveillance hospital: Zhoushan hospital. All isolates were collected from 2013 (41 isolates) to 2014 (66 isolates). The 107 isolates were stored at -80°C in trypticase soy broth (TSB) containing 20% glycerol for further analysis.

### Analysis of virulence genes and Kanagawa phenomenon

*Vibrio parahaemolyticus* can be detected by a species-specific marker (*tlh*) with the pathogenic strains carrying an additional *tdh* and/or *trh* gene. In addition, a distinctive eighth open reading frame of the filamentous phage f237, *orf8*, has been suggested as an useful genetic marker for identification of those pathogenic strains [13–15].

Determination of *tlh tdh*, and *trh* genes as well as the marker (*orf8*) for the *V*. *parahaemolyticus* isolates was performed using multiplex polymerase chain reaction (PCR) according to the methods of Ward and Bej [16] (Table 1). The pathogenic group was defined as *tdh*+ and/or *trh*+; all other isolates were assigned to the non-pathogenic group [15, 17].

**Table 1:** Oligonucleotide primers used for polymerase chain reactions [16].

Target genes	Primers (Probes)	Sequence (5’-3’)	Amplicon Size (bp)
*tlh*	F-tl	AAAGCGGATTATGCAGAAGCACTG	450
	R-tl	GCTACTTTCTAGCATTTTCTCTGC	
	P-tl952	AAGAACTTCATGTTGATGACACT	
*tdh*	F-*tdh*170DG	GTAAAGGTCTCTGACTTTTGGAC	229
	R-*tdh*403	CTACAGAATCATAGGAATGTTGAAG	
	P-*tdh*-341R	ATTTTACGAACACAGCAGAATGA	
*trh*	F-*trh*82	CCATCAATACCTTTTCCTTCTCC	207
	R-*trh*287c	ACCGTCATATAGGCGCTTAAC	
	P-*trh*275	TATTTGTCGTTAGAAATACAACAAT	
*orf8*	F-O3MM824	AGGACGCAGTTACGCTTGATG	369
	R-O3MM1192	CTAACGCATTGTCCCTTTGTAG	
	P-ORFORF8-853	AAGCCATTAACAGTTGAAGGCGTTGACT	

Analysis of the Kanagawa phenomenon produced by the *V*. *parahaemolyticus* isolates was conducted using Wagatsuma blood agar (OXOID, Cambridge, UK) according to China National Food Safety Standard methods, GB 4789.7–2013. Each *V*. *parahaemolyticus* isolate was cultured on a plate of tryptic soy agar (OXOID, Cambridge, UK) containing 3% NaCl for 18 h and then spotted on a Wagatsuma agar plate. The Wagatsuma agar plates were incubated for 18–24 h at 37°C and examined for Kanagawa phenomenon within 24 h.

### Serotyping

Serological analysis of lipopolysaccharide (O) and capsular (K) antigens of the *V*. *parahaemolyticus* isolates was identified by performing agglutination tests using a commercial *V*. *parahaemolyticus* antiserum test kit (Denka Seiken, Tokyo, Japan) according to the manufacturer’s instructions.

### Pulsed-field gel electrophoresis (PFGE)

The PFGE analysis of the *V*. *parahaemolyticus* isolates was performed according to the PulseNet protocol (http://www.pulsenetinternational.org). Briefly, using *Not*I enzyme to restrict the chromosomal DNA. The restriction fragments were resolved with 1% seakem gold agarose gel in 0.5% Tris-boric acid-EDTA buffer using a CHEF Mapper XA system (Bio-Rad Laboratories, Richmond, Calif., USA). The *Xba*I digested DNA from *Salmonella enterica* serotype Braenderup strain H9812 was used as a molecular size marker. The PFGE patterns were analyzed using BioNumerics software (Applied Maths, Belgium). Clustering was performed using the unweighted pair group method and the Dice correlation coefficient with a position tolerance of 1.5%. Clusters were defined on the basis of a 90% similarity cutoff [18].

### Antimicrobial susceptibility testing

The antimicrobial susceptibility of the 107 clinical *V*. *parahaemolyticus* isolates was tested using the broth microdilution method. The results of antimicrobial susceptibility were interpreted as sensitive, intermediate, or resistant, according to the Clinical and Laboratory Standards Institute (CLSI) breakpoint for *Vibrio* species (not *V*. *cholerae*). *Escherichia coli* ATCC 25922 was used as a control. The results were analyzed using the CLSI breakpoints.

## Results

### Distribution of pathogenic and non-pathogenic strains

All of the 107 clinical isolates (100%, 107/107) were positive for the *tlh* gene, among which there were 94 isolates (87.9%, 94/107) carrying the *tdh* gene, and only 4 isolates (3.7%, 4/107) carrying the *trh* gene. In addition, 12 isolates (11.2%, 12/107) isolated from the diarrheal patients were non-pathogenic strains containing neither the *tdh* gene nor the *trh* gene, whereas 92 (86.0%, 92/107) of the tested isolates were classified as pathogenic strains.

28 isolates (26.2%, 28/107) carried the *orf8* gene. 12 isolates (11.2%, 12/107) were determined to carry no *tdh*, *trh*, or *orf8* genes. Analysis of the Kanagawa phenomenon (KP) and molecular detection of virulence genes by PCR of the 107 *V*. *parahaemolyticus* isolates revealed high percentages of clinical isolates producing KP (80/107, 74.8%) and carrying the *tdh* gene (87.9%, 94/107). Among them, 79 isolates (73.8%, 79/107) were positive for both KP and the *tdh* gene and 3 isolates (2.8%) were positive for both KP and the *trh* gene.

### Serotyping

**Fig 1:**
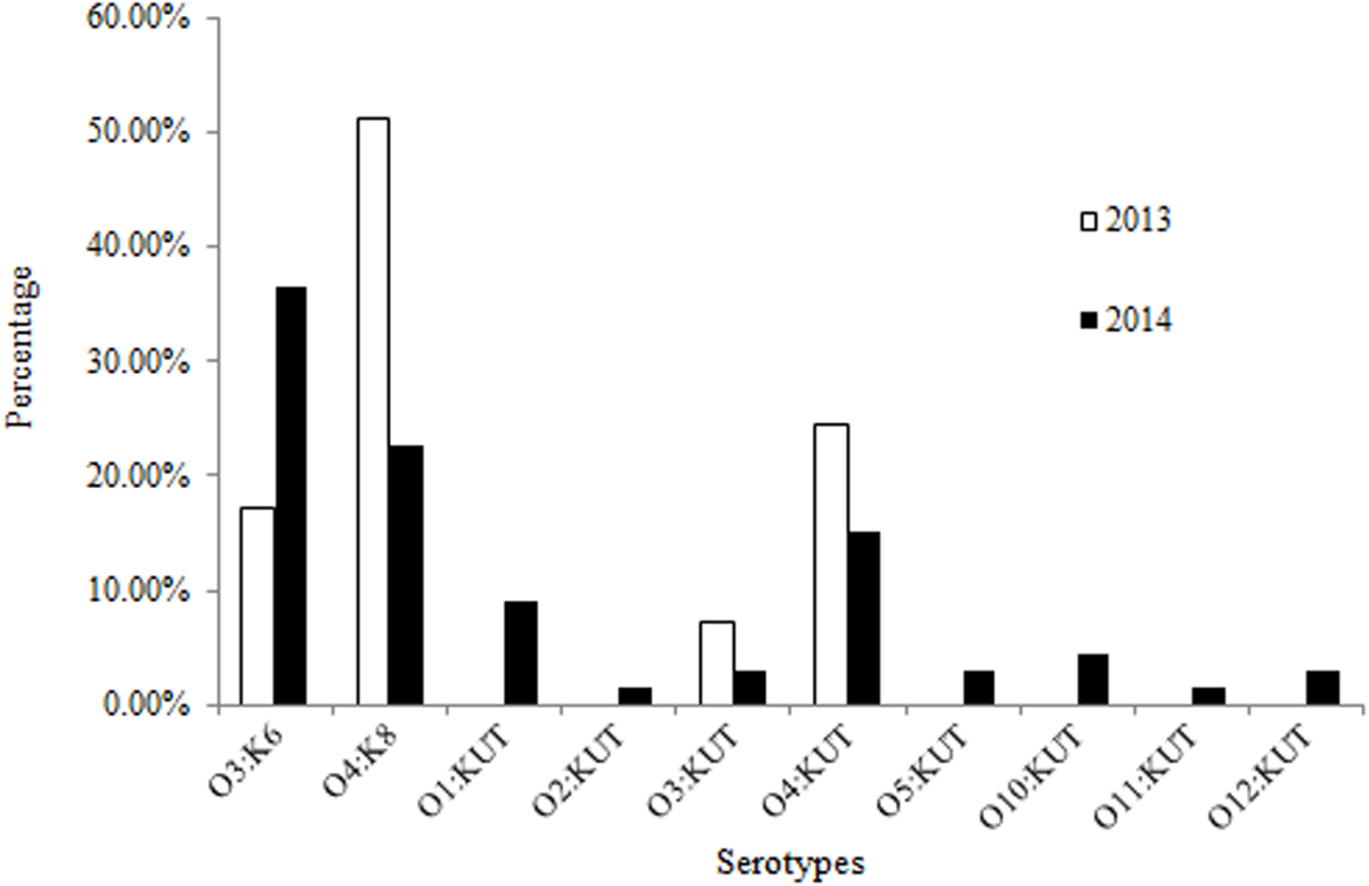
Serological analysis of clinical *V*. *parahaemolyticus* isolates from patients in Zhoushan in 2013 (n = 41) and 2014 (n = 66).

Serological analysis of the 107 *V*. *parahaemolyticus* isolates revealed a total of 10 serovars with O4:K8 (33.6%, 36/107) being the most common one followed by O3:K6 (29.0%, 31/107). These results indicate an increase in the occurrence of O3:K6 strains from 17.1% (7/41) in 2013 to 36.4% (24/66) in 2014 (Fig 1). However, the occurrence of the O4:K8 strains decreased from 51.2% (21/41) in 2013 to 22.7% (15/66) in 2014.

Although all of the 107 isolates were each identified for its O antigen, 40 isolates with 8 (O) serotypes could not be serotyped for K antisera [O4:KUT (20), O1:KUT (6), O3:KUT (5), O10:KUT (3), O5:KUT (2), O12:KUT (2), O2:KUT (1), and O11:KUT (1)]. Among them, only two serotypes (O3:KUT and O4:KUT) were detected in the 2013 isolates, while all of the 8 serotypes were observed in the 2014 isolates (Table 2).

**Table 2:** Serotypes and virulence factors of 107 clinical *V*. *parahaemolyticus* isolated from patients in Zhoushan between 2013 and 2014.

O serogroups	Serovars	No. of isolate (s)	Virulence index			
			*tdh*	*trh*	*orf8* ^a^	Kanagawa phenomenon
O1	O1:KUT	1	+	+	−	+
		1	+	−	−	+
		4	−	−	−	−
O2	O2:KUT	1	−	−	−	+
O3	O3:K6	18	+	−	+	+
		4	+	−	+	−
		8	+	−	−	+
		1	+	−	−	−
	O3:KUT	2	+	−	−	+
		3	−	−	−	−
O4	O4:K8	2	+	−	+	+
		32	+	−	−	+
		2	+	−	−	−
	O4:KUT	2	+	−	+	+
		1	+	−	+	−
		10	+	−	−	+
		7	+	−	−	−
O5	O5:KUT	2	−	−	−	−
O10	O10:KUT	2	+	+	−	+
		1	−	−	−	−
O11	O11:KUT	1	−	+	−	−
O12	O12:KUT	1	+	−	+	+
		1	−	−	−	−

^a^ Associated with pandemic isolates.

### PFGE and cluster analysis

**Fig 2:**
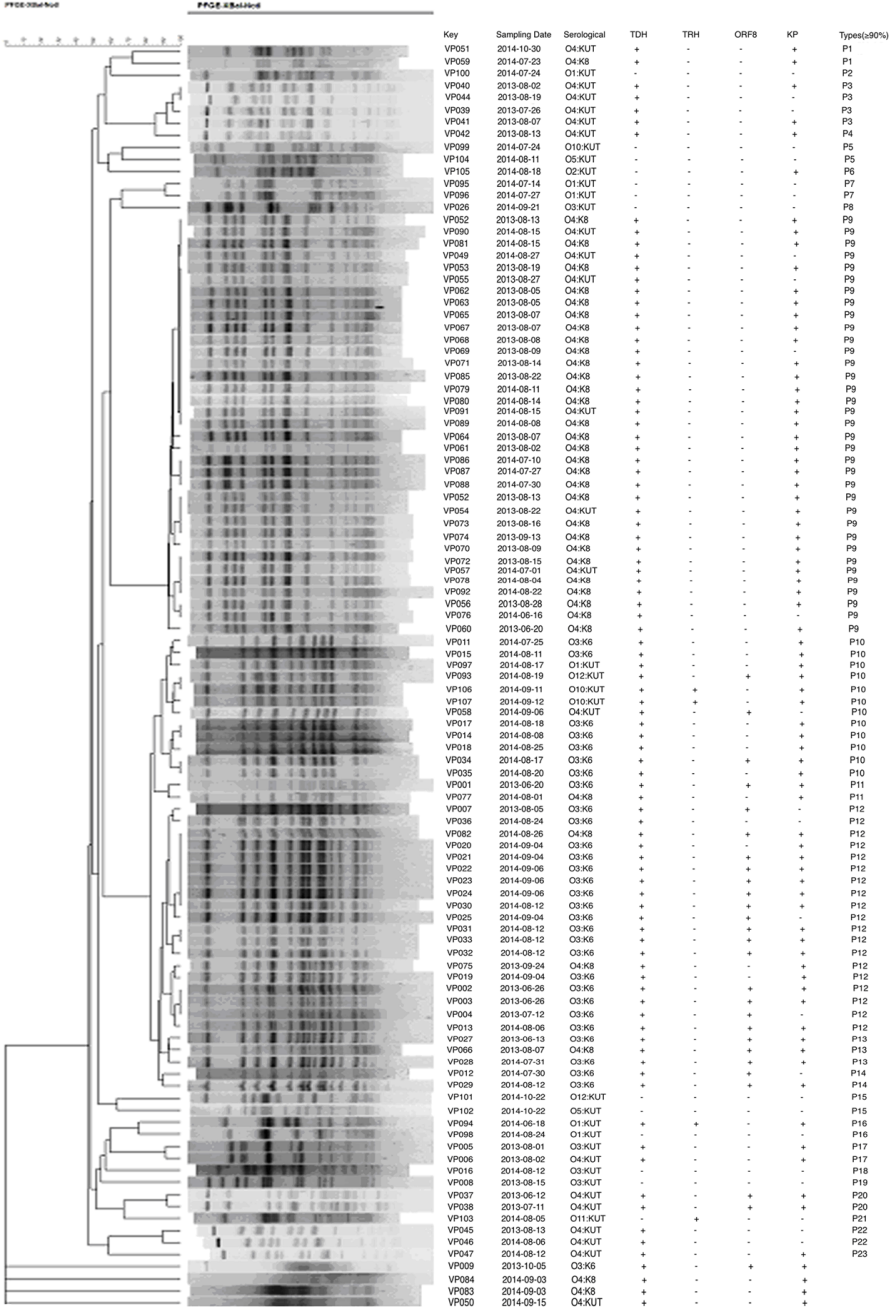
Pulsed-field gel electrophoresis of NotI-digested genomic DNA of selected clinical and foodborne *V*. *parahaemolyticus* isolates obtained in Zhoushan. Strain identification number, isolation date, and seromarkers.

Analysis of the genotypes of the 107 clinical *V*. *parahaemolyticus* isolates found that 4 of the isolates were non-typable due to DNA degradation during endonuclease digestion. PFGE analysis of the remaining 103 isolates yielded 23 distinguishable patterns at a 90% similarity threshold (Fig 2). Among them, 35 isolates were clustered in pattern 9, which contained isolates belonging to two different serotypes, O4:K8 and O4:KUT. Furthermore, the isolates, which were O3:K6 serotype were not all included in the same group, such as VP012 and VP029 were clustered in pattern 14 and the other O3:K6 isolates were clustered in pattern 10, 11, 12 and 13.

### Antimicrobial resistance profile

The antimicrobial susceptibilities of *V*. *parahaemolyticus* are listed in Table 3. The largest proportion of *V*. *parahaemolyticus* strains was resistant to ampicillin (85.5%, 91/106). More than half of the isolates exhibited intermediate levels of susceptibility to cefazolin (54.7%, 58/106). Additionally, more than 94% of the isolates were sensitive to other antimicrobials including gentamicin, amikacin, meropenem, imipenem, cefoxitin, cefepime, ceftazidime, trimethoprim/sulfamethoxazole, chloramphenicol, amoxicillin/clavulanic acid, ciprofloxacin, levofloxacin and tetracycline.

**Table 3:** Antimicrobial susceptibility testing of 107 *V*. *parahaemolyticus* strains.

Antimicrobial agent	Susceptible (%)	Intermediate (%)	Resistant (%)
Gentamicin	94.4	4.7	0.9
Amikacin	96.2	3.8	0.0
Meropenem	100.0	0.0	0.0
Imipenem	100.0	0.0	0.0
Cefoxitin	100.0	0.0	0.0
Cefepime	99.1	0.9	0.0
Ceftazidime	100.0	0.0	0.0
Cefazolin	45.3	54.7	0.0
Trimethoprim/sulfamethoxazole	100.0	0.0	0.0
Chloramphenicol	100.0	0.0	0.0
Amoxicillin/clavulanic acid	100.0	0.0	0.0
Ampicillin	0.0	14.2	85.8
Ciprofloxacin	100.0	0.0	0.0
Levofloxacin	100.0	0.0	0.0
Tetracycline	100.0	0.0	0.0

## Discussion

*V*. *parahaemolyticus* has emerged as an important public health concern worldwide as a pathogen that causes gastroenteritis related to the consumption of various seafood, including crab, shrimp, lobster, fish, and oysters [19]. In the present study, 107 clinical *V*. *parahaemolyticus* strains were collected from 2013 to 2014 in Zhoushan, we identified and analyzed the virulence genes, serotypes, molecular typing by PFGE, and antimicrobial resistance profiles of these clinical strains.

As shown by epidemiological investigation, thermostable direct hemolysin (TDH) is recognized as one of the major pathogenic factors in *V*. *parahaemolyticus*, and is present in 92% to 95% clinical isolates [19, 20], and it contributes to the formation the “Kanagawa phenomenon” (KP) [19], which has been regarded as an important indicator for the identification of pathogenic and non-pathogenic strains [19]. In the present study, among 94 *tdh*+ clinical strains, 15 strains did not show KP, moreover, we found one clinical strain (1/107) that tested positive for KP, though it carried neither *tdh* nor *trh*. Therefore, the KP is not a reliable indicator for identifying pathogenic and non-pathogenic *V*. *parahaemolyticus* strains [19].

As shown in Table 2, 87.9% of the strains carried *tdh*, and only 4 strains carried *trh*, that is, 91.5% of the strains carried *tdh* and/or *trh* gene. This is similar to a previous study reporting 92.5% of the clinical isolates of *V*. *parahaemolyticus* collected in Shanghai as carrying the *tdh* and/or *trh* gene [20]. Furthermore, 11.2% of clinical strains from the diarrheal patients contained neither the *tdh* nor the *trh* gene, which is similar to a previous study reporting 11.8% of clinical isolates of *V*. *parahaemolyticus* collected in Jiangsu province as carrying neither the *tdh* gene nor the *trh* gene [15]. It has been reported that there were outbreaks caused by strains lacking of *tdh* and/or *trh* [21]. Although other pathogenic factors have been recognized, such as type III secretion system 1 and 2 and biofilm formation, the understanding of pathogenicityis still incomplete [21]. Meanwhile, 28 strains contained *orf8*. The *orf8* is suggested to be a marker for detecting recent serotype O3:K6 and other pandemic group strains [16], which were considered as a good target for detection of pathogenic pandemic *V*. *parahaemolyticus* O3:K6 serotype strains. As shown in the current study, the hemolysin genes (*tdh* and *trh*) and *orf8* were tested, furthermore, more pandemic markers, such as *toxRS*/new and type III secretion system could be analyzed to reveal comprehensive pathogenic and pandemic characteristics of clinical *V*. *parahaemolyticus* strains in Zhoushan.

Since *V*. *parahaemolyticus* was discovered 60 years ago [22], it has been considered as a major cause of foodborne illnesses all over the world with serotype O3:K6 being firstly isolated in 1996 in India and subsequently worldwide [15, 23]. In this study, 31 (29%) of 107 clinical *V*. *parahaemolyticus* isolates collected over 2 years from 2013 to 2014 were identified as O3:K6, which is lower than the previous study reporting 54%, 62% and 66% of the clinical isolates of *V*. *parahaemolyticus* collected in Guangdong, Shanghai and Jiangsu respectively [15, 20, 24]. These results demonstrate that *V*. *parahaemolyticus* strains with different serotypes can share common molecular types, therefore, the serotyping was of limited use because there was no match between serotype and PFGE cluster. PFGE has been used as the “gold standard” method to assess genetic diversity and clonal relatedness among *V*. *parahaemolyticus* isolates from different sources [18, 20, 25].

Antibiotics can be used to treat *V*. *parahaemolyticus* infection, and they should be based on the antimicrobial susceptibilities of *V*. *parahaemolyticus*. In the present study, most isolates were sensitive to the antimicrobial agents recommended by CLSI for *Vibrio* species (not V. cholerae) except ampicillin (85.8%). Similar findings have been reported by Chen et al (87.1%) [17]. As shown in Table 3, 100% of the clinical strains in the present study were susceptible to Meropenem, Imipenem, Cefoxitin, Ceftazidime, Trimethoprim/sulfamethoxazole, Chloramphenicol, Amoxicillin/clavulanic acid, Ciprofloxacin, Levofloxacin and Tetracycline.

To the best of our knowledge, this is the first study to investigate virulence factors, serotypes, genotypes, and antimicrobial susceptibility of the clinical strains of *V*. *parahaemolyticus* collected in Zhoushan over 2 years through a continuous surveillance of *V*. *parahaemolyticus* isolated from patients during warm months. Continuous monitoring of *V*. *parahaemolyticus* strains in Zhoushan is necessary to generate information useful for the control of foodborne illnesses caused by *V*. *parahaemolyticus*.

## Conclusions

This study analyzed characteristics of *V*. *parahaemolyticus* strains in diarrheal patients in Zhoushan. Different serotypes were detected in the clinical isolates. Although the clinical *V*. *parahaemolyticus* strains isolated in Zhoushan are not resistant to most common antimicrobial agents, it will be necessary to keep a close attention to the emergence of antimicrobial resistant strains and strengthen the management of antimicrobials. Therefore, this information is useful for the control and treatment of foodborne illnesses caused by *V*. *parahaemolyticus* in Zhoushan.

## References

[1] Ludeke CH, Gonzalez-Escalona N, Fischer M, Jones JL. Examination of clinical and environmental *Vibrio parahaemolyticus* isolates by multi-locus sequence typing (MLST) and multiple-locus variable-number tandem-repeat analysis (MLVA). Front Microbiol. 2015;6:564 doi: 10.3389/fmicb.2015.00564 ; PubMed Central PMCID: PMC4462150. 26113844 10.3389/fmicb.2015.00564 PMC4462150

[2] Liao Y, Li Y, Wu S, Mou J, Xu Z, Cui R, et al Risk Factors for *Vibrio parahaemolyticus* Infection in a Southern Coastal Region of China. Foodborne Pathog Dis. 2015;12(11):881–6. doi: 10.1089/fpd.2015.1988 ; PubMed Central PMCID: PMC4675456. 26287765 10.1089/fpd.2015.1988 PMC4675456

[3] Zarei M, Maktabi S, Ghorbanpour M. Prevalence of *Listeria monocytogenes*, *Vibrio parahaemolyticus*, *Staphylococcus aureus*, and *Salmonella* spp. in seafood products using multiplex polymerase chain reaction. Foodborne Pathog Dis. 2012;9(2):108–12. doi: 10.1089/fpd.2011.0989 . 22044288 10.1089/fpd.2011.0989

[4] Broberg CA, Calder TJ, Orth K. *Vibrio parahaemolyticus* cell biology and pathogenicity determinants. Microbes and infection / Institut Pasteur. 2011;13(12–13):992–1001. doi: 10.1016/j.micinf.2011.06.013 ; PubMed Central PMCID: PMC3384537. 21782964 10.1016/j.micinf.2011.06.013 PMC3384537

[5] Su YC, Liu C. *Vibrio parahaemolyticus*: a concern of seafood safety. Food Microbiol. 2007;24(6):549–58. doi: 10.1016/j.fm.2007.01.005 . 17418305 10.1016/j.fm.2007.01.005

[6] Xie J, Sun X, Pan Y, Zhao Y. Combining basic electrolyzed water pretreatment and mild heat greatly enhanced the efficacy of acidic electrolyzed water against *Vibrio parahaemolyticus* on shrimp. Food Control. 2012;23(2):320–4. doi: 10.1016/j.foodcont.2011.07.019

[7] Yamamoto A, Iwahori J, Vuddhakul V, Charernjiratragul W, Vose D, Osaka K, et al Quantitative modeling for risk assessment of *Vibrio parahaemolyticus* in bloody clams in southern Thailand. International journal of food microbiology. 2008;124(1):70–8. doi: 10.1016/j.ijfoodmicro.2008.02.021 . 18405992 10.1016/j.ijfoodmicro.2008.02.021

[8] Iwahori J, Yamamoto A, Suzuki H, Yamamoto T, Tsutsui T, Motoyama K, et al Quantitative risk assessment of *Vibrio parahaemolyticus* in finfish: a model of raw horse mackerel consumption in Japan. Risk Anal. 2010;30(12):1817–32. doi: 10.1111/j.1539-6924.2010.01444.x . 20626688 10.1111/j.1539-6924.2010.01444.x

[9] Yoon KS, Min KJ, Jung YJ, Kwon KY, Lee JK, Oh SW. A model of the effect of temperature on the growth of pathogenic and nonpathogenic *Vibrio parahaemolyticus* isolated from oysters in Korea. Food Microbiol. 2008;25(5):635–41. doi: 10.1016/j.fm.2008.04.007 . 18541160 10.1016/j.fm.2008.04.007

[10] Wu Y, Wen J, Ma Y, Ma X, Chen Y. Epidemiology of foodborne disease outbreaks caused by *Vibrio parahaemolyticus*, China, 2003–2008. Food Control. 2014;46:197–202. doi: 10.1016/j.foodcont.2014.05.023

[11] Wang X, Zhang H, Zhang L, Zhong K, Shang X, Zhao Y, et al Assessment on dioxin-like compounds intake from various marine fish from Zhoushan Fishery, China. Chemosphere. 2015;118(0):163–9. doi: 10.1016/j.chemosphere.2014.07.057 . 25180652 10.1016/j.chemosphere.2014.07.057

[12] Xue C GH. Quantitative measurement of seafood *Vibrio parahaemolyticus* in Zhoushan city, Zhejiang province. Disease Surveillance. 2008;23(7):424–6.

[13] Han H, Li F, Yan W, Guo Y, Li N, Liu X, et al Temporal and Spatial Variation in the Abundance of Total and Pathogenic *Vibrio parahaemolyticus* in Shellfish in China. PLoS One. 2015;10(6):e0130302 doi: 10.1371/journal.pone.0130302 ; PubMed Central PMCID: PMC4465338. 26061712 10.1371/journal.pone.0130302 PMC4465338

[14] Vongxay K, Pan Z, Zhang X, Wang S, Cheng S, Mei L, et al Occurrence of pandemic clones of *Vibrio parahaemolyticus* isolates from seafood and clinical samples in a Chinese coastal province. Foodborne Pathog Dis. 2008;5(2):127–34. doi: 10.1089/fpd.2007.0045 . 18370608 10.1089/fpd.2007.0045

[15] Chao G, Jiao X, Zhou X, Yang Z, Huang J, Pan Z, et al Serodiversity, pandemic O3:K6 clone, molecular typing, and antibiotic susceptibility of foodborne and clinical *Vibrio parahaemolyticus* isolates in Jiangsu, China. Foodborne Pathog Dis. 2009;6(8):1021–8. doi: 10.1089/fpd.2009.0295 . 19630509 10.1089/fpd.2009.0295

[16] Ward LN, Bej AK. Detection of *Vibrio parahaemolyticus* in shellfish by use of multiplexed real-time PCR with TaqMan fluorescent probes. Applied and environmental microbiology. 2006;72(3):2031–42. doi: 10.1128/AEM.72.3.2031-2042.2006 ; PubMed Central PMCID: PMC1393209. 16517652 10.1128/AEM.72.3.2031-2042.2006 PMC1393209

[17] Chen Y, Chen X, Yu F, Wu M, Wang R, Zheng S, et al Serology, virulence, antimicrobial susceptibility and molecular characteristics of clinical *Vibrio parahaemolyticus* strains circulating in southeastern China from 2009 to 2013. Clinical microbiology and infection: the official publication of the European Society of Clinical Microbiology and Infectious Diseases. 2016;22(3):258 e9–16. doi: 10.1016/j.cmi.2015.11.003 . 26597222 10.1016/j.cmi.2015.11.003

[18] Tsai SE, Jong KJ, Tey YH, Yu WT, Chiou CS, Lee YS, et al Molecular characterization of clinical and environmental *Vibrio parahaemolyticus* isolates in Taiwan. International journal of food microbiology. 2013;165(1):18–26. doi: 10.1016/j.ijfoodmicro.2013.04.017 . 23685468 10.1016/j.ijfoodmicro.2013.04.017

[19] Wang R, Zhong Y, Gu X, Yuan J, Saeed AF, Wang S. The pathogenesis, detection, and prevention of *Vibrio parahaemolyticus*. Front Microbiol. 2015;6:144 doi: 10.3389/fmicb.2015.00144 ; PubMed Central PMCID: PMC4350439. 25798132 10.3389/fmicb.2015.00144 PMC4350439

[20] Zhang H, Sun S, Shi W, Cui L, Gu Q. Serotype, virulence, and genetic traits of foodborne and clinical *Vibrio parahaemolyticus* isolates in Shanghai, China. Foodborne Pathog Dis. 2013;10(9):796–804. doi: 10.1089/fpd.2012.1378 . 23988077 10.1089/fpd.2012.1378

[21] Mahoney JC, Gerding MJ, Jones SH, Whistler CA. Comparison of the pathogenic potentials of environmental and clinical *Vibrio parahaemolyticus* strains indicates a role for temperature regulation in virulence. Applied and environmental microbiology. 2010;76(22):7459–65. doi: 10.1128/AEM.01450-10 ; PubMed Central PMCID: PMC2976215. 20889774 10.1128/AEM.01450-10 PMC2976215

[22] Shinoda S. Sixty Years from the Discovery of *Vibrio parahaemolyticus* and Some Recollections. Biocontrol science. 2011;16(4):129–37. . 22190435 10.4265/bio.16.129

[23] de Jesus Hernandez-Diaz L, Leon-Sicairos N, Velazquez-Roman J, Flores-Villasenor H, Guadron-Llanos AM, Martinez-Garcia JJ, et al A pandemic *Vibrio parahaemolyticus* O3:K6 clone causing most associated diarrhea cases in the Pacific Northwest coast of Mexico. Front Microbiol. 2015;6:221 doi: 10.3389/fmicb.2015.00221 ; PubMed Central PMCID: PMC4371747. 25852677 10.3389/fmicb.2015.00221 PMC4371747

[24] Li B, Luo J, Tan H, Ke B, He D, Ke C, et al Phenotypic and phylogenetic analysis of *Vibrio parahaemolyticus* isolates recovered from diarrhea cases in Guangdong Province, China. International journal of food microbiology. 2015;200:13–7. doi: 10.1016/j.ijfoodmicro.2014.12.009 ; PubMed Central PMCID: PMC4667719. 25662708 10.1016/j.ijfoodmicro.2014.12.009 PMC4667719

[25] Pazhani GP, Bhowmik SK, Ghosh S, Guin S, Dutta S, Rajendran K, et al Trends in the epidemiology of pandemic and non-pandemic strains of *Vibrio parahaemolyticus* isolated from diarrheal patients in Kolkata, India. PLoS Negl Trop Dis. 2014;8(5):e2815 doi: 10.1371/journal.pntd.0002815 ; PubMed Central PMCID: PMC4006737. 24786538 10.1371/journal.pntd.0002815 PMC4006737

